# Breaking barriers with tofersen: Enhancing therapeutic opportunities in amyotrophic lateral sclerosis

**DOI:** 10.1111/ene.16140

**Published:** 2023-11-17

**Authors:** Aniket Saini, Pooja A. Chawla

**Affiliations:** ^1^ Department of Pharmaceutical Analysis ISF College of Pharmacy Moga Punjab India

**Keywords:** ALS, adverse drug reactions, amyotrophic lateral sclerosis, clinical trial, FDA‐approved drugs, tofersen

## Abstract

**Background and purpose:**

Amyotrophic lateral sclerosis (ALS) is a progressive neurodegenerative disease that primarily affects adults, characterized by muscle weakness resulting from the specific death of motor neurons in the spinal cord and brain. The pathogenesis of ALS is associated with the accumulation of mutant superoxide dismutase 1 (SOD1) proteins and neurofilaments in motor neurons, highlighting the critical need for disease‐modifying treatments. Current therapies, such as riluzole and edaravone, provide only symptomatic relief. Recently, tofersen gained approval from the US FDA under the brand name Qalsody as the first and only gene therapy for ALS, addressing a significant pathological aspect of the disease.

**Methods:**

We carried out a literature survey using PubMed, Scopus, National Institutes of Health, and Biogen for articles published in the English language concerned with “amyotrophic lateral sclerosis”, pathophysiology, current treatment, treatment under clinical trial, and the newly approved drug “tofersen” and its detailed summary.

**Results:**

A comprehensive review of the literature on the pathophysiology, available treatment, and newly approved drug for this condition revealed convincing evidence that we are now able to better monitor and treat ALS.

**Conclusions:**

Although treatment of ALS is difficult, the newly approved drug tofersen has emerged as a potential therapy to slow down the progression of ALS by targeting SOD1 mRNA, representing a significant advancement in the treatment of ALS.

## INTRODUCTION

Amyotrophic lateral sclerosis (ALS), also known as Charcot's disease or motor neuron disease, is a typical neuron degenerative condition defined by the gradual decline of motor neurons in the brain and spinal cord. The neurologist Jean‐Martin Charcot first identified the syndrome in 1869 [1, 2]. In ALS there is a specific loss of motor neurons which leads to subsequent paralysis and ultimately results in the death of the affected individual [3]. In adults there is onset of progressive malfunction and subsequent death of upper motor neurons (UMN) and lower motor neurons (LMN) in the motor cortex, brainstem, and spinal cord in ALS. Progressive bulbar palsy (PBP) is another name given to the disorder as the first indications appear in skeletal muscles with bulbar innervation. While primary lateral sclerosis is mainly a UMN manifestation, progressive muscular atrophy (PMA) manifests as dysfunction of LMN specifically affecting skeletal muscles in the trunk or limbs. All four limbs and the bulbar region suffer weakness and muscular atrophy as a result of ALS [2, 4, 5, 6]. The initial weakening in ALS typically initiates in the limb muscles, particularly in the distal muscles rather than the proximal ones [7, 8]. ALS is specifically selective for motor neurons, and mainly spares cognitive, sensory, and autonomic nervous system functions. The only skeletal muscles left untouched are those that control eye movement and the pelvic region [3, 9]. Although the rate of disease development varies, most patients die from neuromuscular respiratory distress within 2–3 years of their initial symptoms [7, 10]. Around 10% of ALS cases are categorized as familial, whereas 90% are believed to be sporadic [11, 12]. In most countries, ALS is a very uncommon condition that affects 1–2 persons per 100,000 individuals annually; the prevalence of the disease is around 5 cases per 100,000 individuals, which illustrates the rapid mortality of the condition. According to statistics from the USA and UK, ALS kills more than 1 in 500 individuals [1, 11]. ALS is believed to arise from a combination of different factors including genetic, environmental, and malfunctions associated with the aging process [13].

In 1993, the gene for Cu/Zn superoxide dismutase 1 (SOD1) was identified as the first gene linked to ALS, responsible for 20% of familial ALS (fALS) cases and 1% to 2% of sporadic ALS (sALS) cases. SOD1 has a redox potential and can convert the superoxide anion into hydrogen peroxide (H_2_O_2_) [2, 7, 14]. Moreover, since 1993, more than 200 different mutations of *SOD1* have been reported. Of these mutations, 108 are missense mutations which affect 66 codons in which one amino acid is exchanged with another while the full length of the polypeptide remains the same. These missense mutations are regarded as the root cause of ALS. Of 11 nonsense mutations which shorten the length of the polypeptide, only two mutations have been associated with the disorder. Six mutations are silent DNA variants in which the sequence and length of the polypeptide do not change. These allelic variations are probably not the cause of ALS [2, 15]. It is still unclear exactly how *SOD1* contributes to the pathogenesis of ALS. New research has revealed that the SOD1 protein, which is associated with ALS, may have additional functions beyond its known role as an antioxidant enzyme. Recent findings indicate that *SOD1* could play crucial roles in activating the transcription of nuclear genes and acting as an RNA‐binding protein. These newly identified functions are highly significant in understanding the underlying mechanisms of ALS and its development [15, 16]. The aggregation of SOD1 protein, forming insoluble structures, disrupts various cellular processes that are detrimental to neurons and contributes to their death in ALS. These abnormal processes include mitochondrial malfunction, stress in endoplasmic reticulum, and problems with protein degradation. In individuals with ALS who have *SOD1* mutations, accumulations of SOD1 inclusions specifically occur in the anterior motor neurons located in the spinal cord [16, 17]. On April 25, 2023, the US Food and Drug Administration (FDA) approved tofersen under the brand name Qalsody for the treatment of ALS in adults with a *SOD1* gene mutation. Tofersen (BIIB067), an antisense oligonucleotide, targets the SOD1 gene in individuals with ALS caused by SOD1 mutations. The specific mechanism of action of tofersen involves modulating the production of SOD1 protein. Tofersen is a synthetic, single‐stranded DNA molecule that is designed to bind to the messenger RNA (mRNA) derived from the *SOD1* gene. By targeting the SOD1 mRNA, tofersen works through an antisense mechanism. It binds to the mRNA sequence and triggers a process known as RNase H‐mediated degradation. This degradation prevents the translation of SOD1 mRNA into SOD1 protein, thereby lowering the amount of mutant SOD1 protein in affected cells [18].

## PATHOPHYSIOLOGY OF ALS

The precise mechanisms of neuron degeneration in ALS remain unknown; however, several cellular and molecular processes have been connected to disease development. These include mitochondrial dysfunction, problems with axonal transport, toxic protein aggregates, impaired protein breakdown involving autophagy or proteasome (or both), the spread of pathological proteins in a prion‐like manner, oxidative stress, increased metabolism, excitotoxicity, decreased support from non‐neuronal cells in terms of neurotrophic factors, inflammation, defects in RNA metabolism, and RNA toxicity [19].

There have been more than 20 identified genes that contribute to the development of ALS or increase the risk of disease development. In European populations, four specific genes (*C9orf72*, *SOD1*, *TARDBP*, and *FUS*) are responsible for causing the disease in up to 70% of individuals diagnosed with fALS. These genes have been found to have a substantial influence on the development and progression of ALS within these populations. Over 170 identified mutations associated with ALS have been found in different regions of the 153‐amino acid *SOD1* polypeptide. Interestingly, many of these mutated variants still exhibit partial or even full dismutase activity. Surprisingly, there is no clear relationship between the decrease in enzymatic activity and the age at which the disease starts or how quickly it progresses. As a result of these discoveries, researchers have concluded that a decline in *SOD1* dismutase activity is not the only primary contributor to the onset of ALS. Instead, it is thought that the disease is caused by one or more toxic characteristics displayed by the different mutant versions of *SOD1*. These toxic properties could arise from structural changes, altered interactions with other cellular components, or aggregate generation that disrupts normal cellular processes and leads to neuron degeneration [1, 20, 21]. The main pathways for ALS pathophysiology are described in the following sections.

### Mutation in *SOD1* gene

ALS is associated with a mutation in the *SOD1* gene which results in the toxic gain of function. This mutation in the *SOD1* gene causes the formation of mutant SOD1 proteins. These mutated SOD1 proteins accumulate within the motor neurons and glial cells, leading to protein aggregation and the formation of toxic protein clumps, and cause dysfunction in the axonal transport system. How exactly these intracellular aggregates are toxic to motor neurons remains unclear, although several possible cytotoxic mechanisms have been proposed, including;

- Coaggregation with vital cellular constitutes,
- Inhibition of normal proteosomic function, and
- Mechanical or biochemical effects on the cell, such as the disruption of axonal transport systems [1, 22, 23].

### Oxidative stress

The accumulated SOD1 proteins, together with other factors such as accumulation of Ca^+^ ions, can contribute to oxidative stress within the motor neurons. This oxidative stress can lead to mitochondrial dysfunction and neuronal cell death through neuroinflammation [1, 24, 25].

### Glutamate excitotoxicity

**FIGURE 1:**
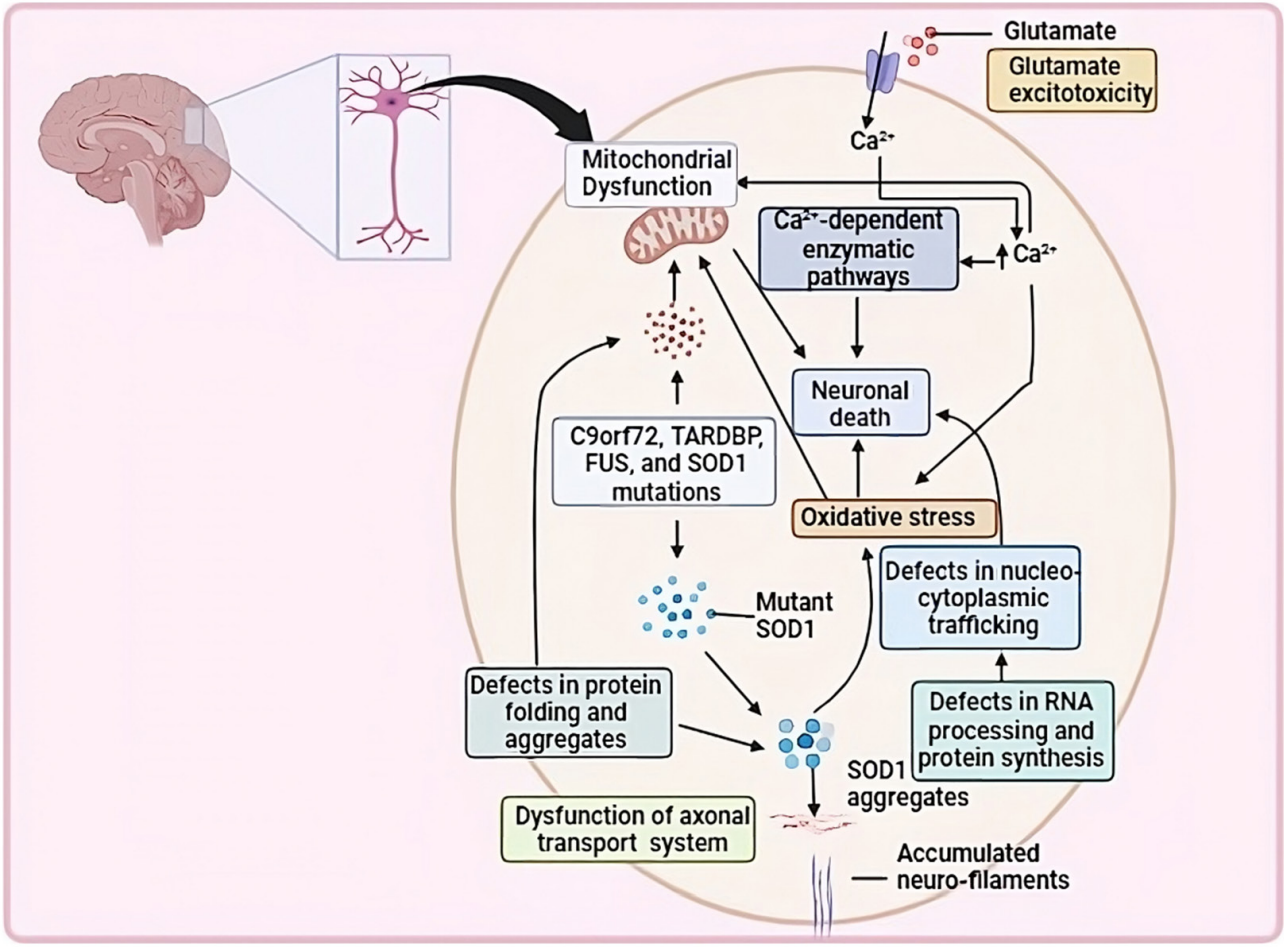
Pathophysiology of amyotrophic lateral sclerosis.

In recent decades, studies have suggested that glutamate excitotoxicity has a potential role in the pathogenesis of ALS. Glutamate excitotoxicity is mediated by excessive activation of postsynaptic receptors. On the postsynaptic side there is increased expression of NMDA receptors, which are permeable to influx of Ca^2+^ ions, resulting in increased intracellular Ca^2+^ concentration and activation of Ca^2+^‐dependent enzymatic pathways that mediate neuronal death. Glutamate excitotoxicity may also result in production of free radicals that can further damage intracellular organelles and thereby cause cell death. The pathophysiology of ALS is illustrated in Figure 1 [1, 26, 27].

## CURRENTLY AVAILABLE TREATMENTS FOR ALS

Currently, there are only two drugs that have been officially approved and labeled for the treatment of ALS, namely riluzole and edaravone. However, it is important to note that edaravone is only available in a limited number of countries globally. Specifically, it is accessible in the USA, Japan, Canada, and Switzerland within Europe. Key details such as structure, mechanism of action, dosage, and side effects of the two currently available drugs are listed in Table 1 [4, 28, 29]. Although the cost of tofersen is high compared with existing drugs, it offers many advantages.

**TABLE 1:** Currently available treatments for amyotrophic lateral sclerosis.

Drug	Structure	Mechanism of action	Dosage	Side effects	Reference
Riluzole	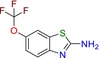	Riluzole is a glutamate antagonist. The exact mechanism is not completely understood. Pharmacological properties that may be related to its effects: Inhibiting the release of glutamate Inactivating voltage‐dependent sodium channels Capacity to interfere with intracellular processes that follow transmitter binding at excitatory amino acid receptors	Oral tablets. Recommended dose 50 mg/12 h	Most commonly observed: dose‐related nausea, asthenia, gastrointestinal problems, and elevated liver enzyme levels	[28]
Edaravone	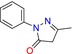	Edaravone is thought to eliminate hydroxyl radicals and lipid peroxides by acting as a scavenger of free radicals and reactive oxygen species [6]. While its mechanism of action in ALS remains unclear, edaravone is thought to act to decrease oxidative damage to neurons, especially motor neurons and nearby glial cells that are prone to damage in ALS. Drug company officially states that the mechanism of action is unknown	Intravenous (IV) infusion. Cycle 1 of the recommended treatment is an IV infusion of 60 mg/day for 14 days, followed by a 14‐day drug‐free period, and then daily dosage for 10/14 days, then another 14‐day drug‐free period (cycles 2–6)	Urine glucose. The level of glucose in the blood exceeds the ability of the kidneys to absorb it	[29]

## OTHER DRUGS UNDER CLINICAL TRIAL FOR THE TREATMENT OF ALS

Certain drugs have been used for the treatment of non‐neurological conditions for many decades and may also possess potential therapeutic effects in neurological disorders. Key details such as structure, trial code, mechanism of action, trial participants, and sponsor for all the drugs currently under clinical trial are listed in Table 2.

**TABLE 2:** Drugs currently under clinical trial for the treatment of amyotrophic lateral sclerosis.

Drug	Structure	Trial code	Mechanism	Participants (*n*)	Sponsor	References
TUDCA	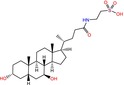	NCT00877604 Phase 2	Neuroprotective activity and protects the cell against the harmful effect of NO toxicity	34	Fondazione IRCCS Istituto Neurologico Carlo Besta	[31]
Tamoxifen	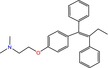	NCT02166944 Phase 1, 2	Also exhibits neuroprotective activity by regulating damage caused by inflammation and promoting autophagy	20	Taipei Medical University Shuang Ho Hospital	[32]
Levosimendan	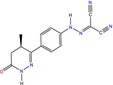	NCT03505021 Phase 3	Selective binding to troponin C, which then sensitizes both fast and slow skeletal muscles and also enhances the efficiency and contractility of the diaphragm	496	Orion Corporation, Orion Pharma	[33]
Masitinib	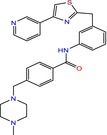	NCT02588677 Phase 2, 3	Ability to protect neurons through its immunomodulatory properties. It specifically targets activated microglia and mast cell activity in both the peripheral and central nervous systems, thereby exerting a neuroprotective effect	394	AB Science	[35]
Deferiprone	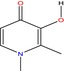	NCT03293069 Phase 2, 3	Has properties of an effective iron chelator and prevents the accumulation of iron in the central motor tract of ALS patients	372	University Hospital, Lille	[36]
Fasudil	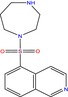	NCT01935518 Phase 2	ROCK‐inhibitor that has shown significant improvement in survival and motor function in mutant SOD1 model when administered presymptomatically	10	Peking University Third Hospital	[37]

Abbreviations: ALS, amyotrophic lateral sclerosis; NO, nitric oxide; ROCK, Rho‐associated protein kinase; SOD1, superoxide dismutase 1.

### TUDCA

Tauro‐ursodeoxycholic acid (TUDCA) is a well‐known medication used by gastroenterologists to treat chronic cholestatic liver and gallstone diseases. It can be taken orally and can cross the blood–brain barrier. TUDCA has been found to have neuroprotective properties in cells that express mutant *SOD1* (specifically, mutations A4V and G93A) which are involved in motor neuron‐neuroblastoma hybrid cells. TUDCA protects these cells against the harmful effects of nitric oxide (NO) toxicity. TUDCA has also gained interest in the context of ALS due to its ability to inhibit apoptosis through its interference with the mitochondrial pathway of cell death. This inhibition leads to a reduction in the production of harmful oxygen radicals, as well as a decrease in endoplasmic reticulum stress and caspase activation. Two phase 2 studies showed promising results in ALS which justified initiating a phase 3 trial [4, 30, 31].

### Tamoxifen

Tamoxifen, a long‐standing approved treatment for breast cancer, acts by selectively influencing estrogen receptors. Apart from its anticancer effects, tamoxifen also exhibits neuroprotective characteristics by regulating damage caused by inflammation and promoting autophagy. To evaluate its potential in treating ALS, a phase 2 study was carried out involving 18 ALS patients who were followed for 12 months. The study employed a randomized, double‐blind, placebo‐controlled approach [4, 32].

### Levosimendan

Levosimendan was originally developed for the treatment of heart failure in the field of cardiology. Its mode of action involves selectively binding to troponin C, which then sensitizes both fast and slow skeletal muscles. Furthermore, intravenous administration of levosimendan has been demonstrated to enhance the efficiency and contractility of the diaphragm in healthy individuals. These promising results regarding diaphragm muscle function led to a study being conducted in individuals with ALS. The study utilized a randomized, double‐blind, placebo‐controlled, crossover design with three periods, followed by an additional 6‐month open‐label follow‐up phase [4, 33, 34].

### Masitinib

Masitinib is an orally administered tyrosine kinase inhibitor that is used for treating mastocytosis. This compound has shown promising results in animal models of ALS with SOD1‐G93A mutations. The interest in using masitinib for ALS stems from its ability to protect neurons through its immunomodulatory properties. It specifically targets activated microglia and mast cell activity in both the central and peripheral nervous systems, thereby exerting a neuroprotective effect.

One notable study among several publications investigating masitinib in ALS was a double‐blind, placebo‐controlled, randomized phase 2/3 study. This study involved 394 patients who were either given riluzole (100 mg/day) plus placebo or masitinib at doses of 4.5 or 3.0 mg/kg/day. The positive results obtained from this study prompted the suggestion that a larger study be conducted. The primary endpoint of the study was to measure the decline in the ALS Functional Rating Scale‐Revised (ALSFRS‐R) score over the course of 48 weeks [4, 35].

### Deferiprone

There is sufficient literature supporting the involvement of iron in neuron degenerative diseases, particularly in ALS. Iron is described as a cofactor for several enzymes involved in the functioning of motor neurons, including mitochondrial aerobic metabolism. Furthermore, studies have shown increased levels of serum iron, ferritin, and transferrin in ALS patients compared to healthy individuals. Postmortem studies have also indicated the accumulation of iron in the central motor tract of ALS patients, which has strengthened the hypothesis of iron's role in ALS.

The FAIRALS study is an ongoing clinical trial conducted by the French ALS Centre of Lille. It is a randomized, double‐blind, placebo‐controlled study that aims to evaluate the effectiveness of the iron chelator deferiprone in the treatment of ALS. The trial is expected to include 372 participants, and the treatment period will span 12 months. Half the participants will be assigned to receive a daily dose of 600 mg deferiprone, while the other half will receive a placebo. The study is registered under the identifier NCT03293069 [4, 36].

### Fasudil

Fasudil is a type of small molecule inhibitor known as a Rho‐associated protein kinase inhibitor (ROCK‐inhibitor). Its initial development focused on its potential application in treating vasospasm that occurs after subarachnoid hemorrhage. It has been found that ROCK plays a crucial role in triggering the signaling pathway for axonal degeneration, which is activated when specific receptors bind to axonal growth inhibitory molecules.

ROCK inhibitors have been extensively investigated in the context of neurodegenerative diseases and have shown both neuroprotective and pro‐regenerative effects in models of Parkinson's disease (PD). In the case of ALS, ROCK inhibitors have shown significant improvements in survival and motor function in the SOD1(G93A) mouse model when administered at a presymptomatic stage. A phase 2a clinical trial is expected to commence soon in ALS, involving multiple centers and employing a randomized, double‐blind, placebo‐controlled, prospective design with the aim of determining the appropriate dosage of the ROCK inhibitor. Fasudil, the ROCK inhibitor of interest, will be administered intravenously twice daily for 20 days of treatment. The trial will consist of three arms: one receiving 30 mg/day fasudil, another receiving 60 mg/day fasudil, and a placebo group serving as a control [4, 37].

## CHEMISTRY OF TOFERSEN

**FIGURE 2:**
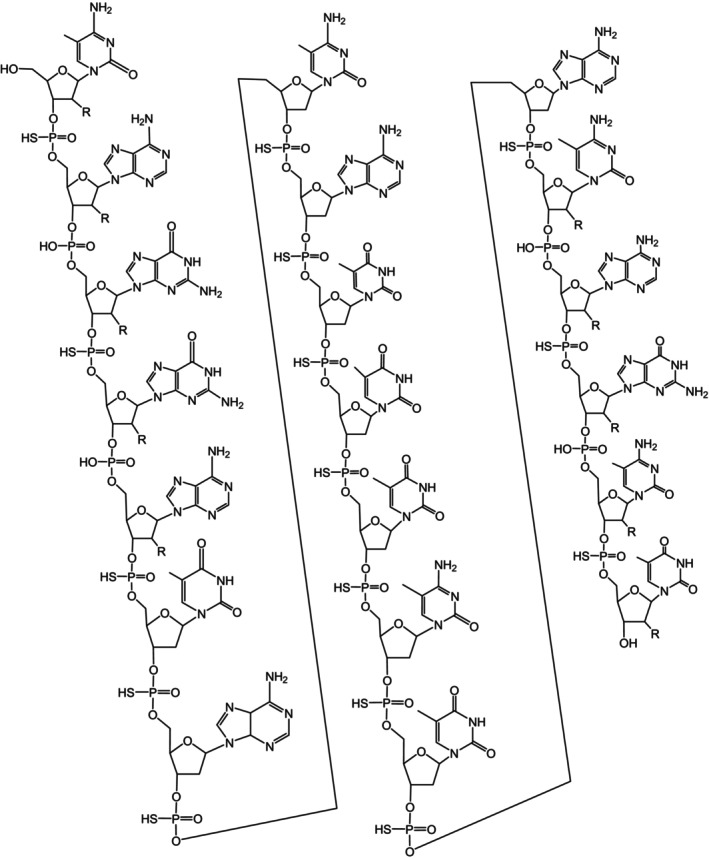
Structure of tofersen.

Tofersen is a 20‐base residue (20‐mer) antisense oligonucleotide with a mixed backbone structure consisting of 5‐10‐5 MOE gapmer. It is composed of 19 inter‐nucleotide linkages, with 15 of them being 3′‐O to 5′‐O phosphorothioate diesters, and the remaining four being 3′‐O to 5′‐O phosphate diesters. Among the 20 sugar residues, 10 are 2‐deoxy‐D‐ribose, while the other 10 are 2′‐O‐(2‐methoxyethyl)‐D‐ribose (MOE). The arrangement of the residues is such that there are five MOE nucleosides positioned at both the 5′ and 3′ ends of the molecule, creating a gap of 10 2′‐deoxynucleosides in between. Additionally, the cytosine and uridine bases within the molecule are methylated at the 5‐position (Figure 2) [38].

## MECHANISM OF ACTION OF TOFERSEN

**FIGURE 3:**
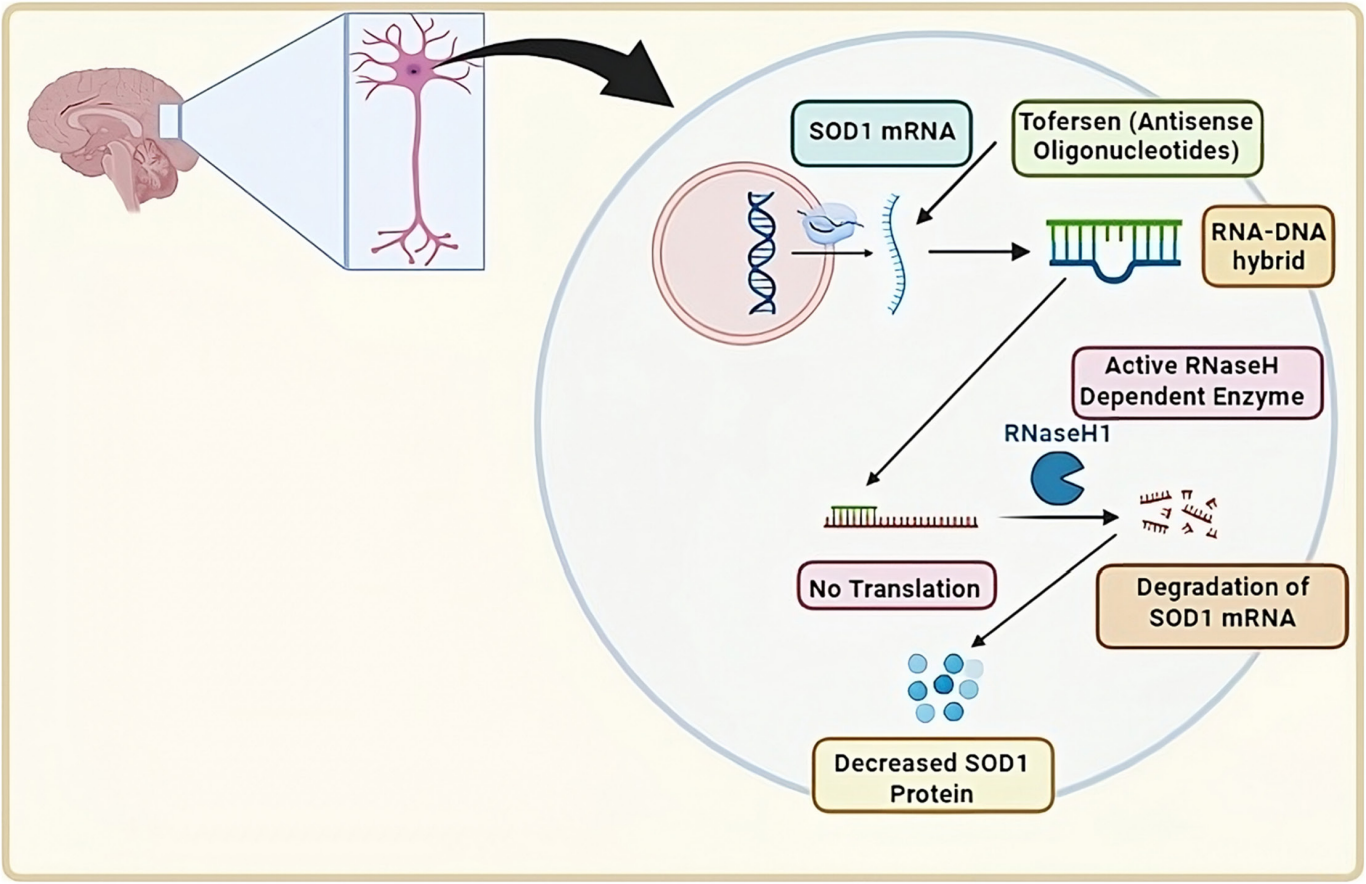
Mechanism of action of tofersen.

Tofersen is an antisense oligonucleotide that has been developed to specifically target SOD1 mRNA. This antisense oligonucleotide utilizes the process of RNase H‐dependent degradation of SOD1 mRNA, resulting in a decrease in the production of SOD1 protein. Tofersen is administered intrathecally, directly into the cerebrospinal fluid (CSF) surrounding the spinal cord. It enters motor neurons and binds specifically to SOD1 mRNA and forms an RNA–DNA hybrid. After forming an RNA–DNA hybrid, tofersen activates RNase H‐dependent enzyme. This RNA–DNA hybrid is a substrate for the RNase H‐dependent hybrid and it cleaves RNA strands resulting in the degradation of mutant SOD1 mRNA. This leads to the reduction of SOD1 mRNA in the motor neurons which ultimately leads to reduction in the mutant SOD1 protein and of neurofilament light chains in plasma, as there are fewer templates of SOD1 mRNA for the translation process to produce mutant SOD1 protein, which causes progression of the disease (Figure 3) [18, 39, 40, 41].

## PHARMACOKINETICS OF TOFERSEN

The pharmacokinetic data for tofersen is comparable in healthy individuals and people with ALS. Tofersen is injected intrathecally into the CSF, allowing it to distribute from the CSF to the tissues of the central nervous system (CNS). The maximum concentration of tofersen in the CSF is achieved with the third dose, which is the final dose of the loading period. Following the loading phase, there is minimal to no accumulation of tofersen in the CSF with monthly dosing after the loading phase. Tofersen is delivered into the systemic circulation from the CSF, and it takes, on average, 2–6 h for it to reach its peak plasma concentration (*T*_max_). With a monthly maintenance dose, tofersen did not accumulate in the plasma. Autopsy examination of CNS tissue from patients (*n* = 3) treated with intrathecal tofersen revealed its distribution within the CNS tissues. Tofersen is neither a substrate for cytochrome P450 (CYP450) enzymes nor is it an inducer or inhibitor of CYP450 enzymes. It is predicted that tofersen is mainly metabolized through exonuclease (3′ and 5′)‐mediated hydrolysis. The primary elimination pathway has not been fully elucidated [38].

## PHARMACODYNAMICS

### Effect of tofersen on total CSF SOD1 protein

In clinical studies, the level of total SOD1 protein was assessed. Clinical Study Part C (NCT02623699) showed that the total CSF SOD1 protein decreased by 35% in the tofersen‐treated group compared to a 2% decline in the placebo group at day 196.

### Effect of tofersen on neurofilament protein

A blood‐based biomarker of axonal damage and neurodegeneration, plasma neurofilament, has been studied in clinical trials. Mean plasma neurofilament was reduced to 55% in the tofersen‐treated group compared to a 12% increase in the placebo group at day 196 of Clinical Study Part C (NCT02623699).

### Cardiac electrophysiology

There is no clinically significant lengthening of the QTc interval at the maximum dose regimen of tofersen [38].

## CLINICAL TRIALS OF TOFERSEN

Various clinical trials for the study of tofersen have been conducted by Biogen. Phase 1–2 studies were conducted involving 50 patients, the primary objective being to evaluate the safety and pharmacokinetic data and to assess the SOD1 concentration change from baseline at day 85. These trials were conducted at different 18 sites in USA, Canada, and four countries in Western Europe (Germany, France, Belgium, and the UK). Five doses of tofersen and a placebo were provided intrathecally over the course of 12 weeks to the participants in each dose cohort (20, 40, 60, and 100 mg) in a ratio of 3:1. Tofersen or placebo was delivered as a single dose on days 1, 15, 29, 57, and 85. A total of 55 participants completed screening between November 7, 2016 and July 17, 2018, and 50 of them were recruited in the phase 1–2 trial. Of the 50 participants, 12 received placebo, 10 received 20 mg, 9 received 40 mg, 9 received 60 mg, and 10 received 100 mg. Up to day 85, participants who received tofersen experienced a reduction in geometric mean ratios of SOD1 protein concentrations from a baseline of 1% in the 20 mg dose group, 27% in the 40 mg dose group, 21% in the 60 mg dose group, and 36% in the 100 mg dose group; participants who received placebo experienced a 3% reduction in the ratio [18].

From March 2019 through July 2021, a phase 3, double‐blind, randomized, placebo‐controlled experiment was carried out. Some 32 sites in 10 different countries approved participants for the clinical trial. The trial included a 28‐day screening period, a 168‐day medication period, and a 28–56‐day follow‐up period. In this trial, a total of 108 participants were enrolled. Of the 108 participants, 72 were assigned to receive tofersen (39 participants expected to have faster progression) and 36 to receive placebo (21 participants expected to have faster progression). In this phase 3 trial, randomly enrolled adults with mutations in SOD1 were assigned to receive eight doses of tofersen (100 mg) or placebo in a 2:1 ratio over a period of 168 days. Intrathecal bolus injections of tofersen (100 mg) or an equivalent amount of a placebo were given to participants via lumbar puncture during a 168‐day period in three doses, once after 7 days, then five doses once after 28 days. In the faster‐progression subgroup, in participants who received tofersen (100 mg) the total concentration of SOD1 protein in CSF was decreased by 29%, as compared with those who received placebo who showed an increase of 16%. In comparison, in the slower‐progression subgroup, participants who received tofersen (100 mg) showed a 40% decrease in the concentration of SOD1 proteins, compared with the participants who received placebo who experienced a 19% reduction (Table 3) [42].

**TABLE 3:** Clinical trials of tofersen.

Phase	Trial code	Participants (*n*)	Purpose of study	Sponsor	Completion date	References
NA	NCT04972487	NA	An early access program to provide access to tofersen to eligible participants with ALS associated with mutations in the SOD1 gene	Biogen	NA	[43]
Phase 1	NCT03764488	8	To assess the safety, tolerability, and distribution in the CNS of a microdose of radiolabeled BIIB067 (99mTc‐MAG3‐BIIB067) co‐administered with BIIB067 (tofersen)	Biogen	July 10, 2021	[44]
Phase 3	NCT02623699	178	A three‐part study to assess the efficacy, safety, tolerability, PK, and PD of tofersen in adult participants with ALS associated with SOD1 mutations	Biogen	July 16, 2021	[18, 45]
Phase 3	NCT03070119	138	To assess the long‐term safety and tolerability of tofersen in participants with ALS and confirmed SOD1 mutations	Biogen	June 13, 2024	[18, 42, 46]
NA	NCT05725759	10	To describe the effect of a personalized rehabilitation program for patients with SOD1 ALS treated with tofersen	Washington University School of Medicine	December 2024	[47]
NA	NCT05852418	25,000	To gather information on the use of assistive devices, medications (tofersen, nusinersen, risdiplam, as well as symptomatic treatments), and other healthcare interventions (e.g., ventilator therapy and nutritional support) in ALS and SMA patients	Ambulanzpartner Soziotechnologie APST GmbH	June 2025	[48]
Phase 3	NCT04856982	150	To assess the effectiveness of tofersen in pre‐symptomatic adult carriers of SOD1 mutations with elevated neurofilament levels	Biogen	August 7, 2027	[49]

Abbreviations: ALS, amyotrophic lateral sclerosis; CNS, central nervous system; NA, not applicable; PD, pharmacodynamics; PK, pharmacokinetics; SMA, spinal muscular atrophy; SOD1, superoxide dismutase 1.

## ADVERSE EFFECTS

The most frequently reported adverse symptoms among participants who received tofersen for the treatment of ALS included post‐lumbar puncture headaches, pain in the arms and legs, procedural pain, falls, and back pain. Additionally, 7% of all participants who received tofersen experienced more severe adverse effects, such as myelitis, chemical or aseptic meningitis, lumbar radiculopathy, increased intracranial pressure, and papilledema [18]. Data from clinical trials indicates that approximately 81% of participants who received tofersen exhibited symptoms associated with lumbar puncture events. Additionally, approximately 46% experienced headaches, about 57% reported procedural pain, 24% had back pain, roughly 26% suffered from pain in their arms or legs, and approximately 10% of participants encountered an elevated white cell count in their CSF [42].

## CONCLUSIONS

ALS is a neurodegenerative disorder that primarily affects adult individuals leading to the degeneration and death of motor neurons in the spinal cord and brain, resulting in the progressive weakening of limb muscles. One important element in the onset of ALS is the accumulation of mutant SOD1 proteins and neurofilaments in motor neurons. As the first and only gene therapy created to target a critical ALS pathogenesis by lowering the quantities of SOD1 mutant proteins in motor neurons, tofersen marks a revolutionary achievement. This medical strategy may be able to slow the course of ALS. When tofersen binds to mutant SOD1 mRNA, RNase H‐dependent enzymes are activated, causing degradation of the mutant mRNA. As a result, this medication significantly reduces the buildup of SOD1 mutant proteins in motor neurons. For tofersen, encouraging pharmacokinetic characteristics have been observed.

## AUTHOR CONTRIBUTIONS

**Pooja A. Chawla:** Conceptualization; supervision; writing – review and editing. **Aniket Saini:** Conceptualization; writing – original draft.

## CONFLICT OF INTEREST STATEMENT

The authors declare no conflicts of interest.

## Data Availability

Data sharing is not applicable to this article as no new data were created or analyzed in this study.
